# Association of chronotype with language and episodic memory processing in children: implications for brain structure

**DOI:** 10.3389/fnint.2024.1437585

**Published:** 2024-08-07

**Authors:** Masatoshi Yamashita, Qiulu Shou, Yoshifumi Mizuno

**Affiliations:** ^1^Research Center for Child Mental Development, University of Fukui, Fukui, Japan; ^2^Division of Developmental Higher Brain Functions, United Graduate School of Child Development, University of Fukui, Fukui, Japan; ^3^Department of Child and Adolescent Psychological Medicine, University of Fukui Hospital, Fukui, Japan

**Keywords:** children, chronotype, language, episodic memory, brain structure

## Abstract

**Introduction:**

Chronotype refers to individual preference in circadian cycles and is associated with psychiatric problems. It is mainly classified into early (those who prefer to be active in the morning and sleep and wake up early) and late (those who prefer to be active in the evening and sleep and wake up late) chronotypes. Although previous research has demonstrated associations between chronotype and cognitive function and brain structure in adults, little is known regarding these associations in children. Here, we aimed to investigate the relationship between chronotype and cognitive function in children. Moreover, based on the significant association between chronotype and specific cognitive functions, we extracted regions-of-interest (ROI) and examined the association between chronotype and ROI volumes.

**Methods:**

Data from 4,493 children (mean age of 143.06 months) from the Adolescent Brain Cognitive Development Study were obtained, wherein chronotype (mid-sleep time on free days corrected for sleep debt on school days) was assessed by the Munich Chronotype Questionnaire. Subsequently, the associations between chronotype, cognitive function, and ROI volumes were evaluated using linear mixed-effects models.

**Results:**

Behaviorally, chronotype was negatively associated with vocabulary knowledge, reading skills, and episodic memory performance. Based on these associations, the ROI analysis focused on language-related and episodic memory-related areas revealed a negative association between chronotype and left precentral gyrus and right posterior cingulate cortex volumes. Furthermore, the precentral gyrus volume was positively associated with vocabulary knowledge and reading skills, while the posterior cingulate cortex volume was positively associated with episodic memory performance.

**Discussion:**

These results suggest that children with late chronotype have lower language comprehension and episodic memory and smaller brain volumes in the left precentral gyrus and right posterior cingulate cortex associated with these cognitive functions.

## 1 Introduction

Chronotype is commonly defined as the individual preferences in the sleep–wake cycle (Zavada et al., 2005; Adan et al., 2012). It is typically classified into three types: morning, evening, and intermediate chronotypes, using a self-assessment tool such as the Munich Chronotype Questionnaire (MCTQ) (Roenneberg et al., 2003). Morning chronotype, also known as early chronotype, refers to the preference to be active in the morning, and sleep and wake early. Evening chronotype individuals, also known as late chronotypes, prefer to be active in the evening, and sleep and wake up late. The intermediate chronotype refers to the lack of a preference for morning or evening. Roenneberg et al. (2004) examined 25,000 children from the MCTQ database and reported that most children are early chronotypes, and tend to shift towards late chronotypes around the age of 20 years. In addition, a recent study of 957 Colombian adolescents (mean age 14.6 years) revealed that late chronotype was associated with higher levels of behavioral problems (i.e., attention and social problems) measured using the Youth Self-Report and Child Behavior Checklist (Zhu et al., 2023). Moreover, late chronotype is associated with an increased risk of psychiatric disorders, such as depression (Antypa et al., 2016; Lunsford-Avery et al., 2021). These findings suggest that chronotype plays an important role in mental health maintenance and is involved in the onset of psychiatric disorders. Additionally, chronotype-related brain structural differences may be associated with mental health issues (Zou et al., 2022). Given the importance of chronotype in mental health and its potential link to brain structure, it is crucial to explore whether chronotype is associated with cognitive function and brain structure. However, little is known about its association with cognitive function and brain structure in children.

Previous studies have focused on the relationship between chronotype and cognitive function in adults. Several studies have found higher intelligence scores, including for memory and processing speed, in late chronotypes (Roberts and Kyllonen, 1999; Gorgol et al., 2020). In addition, late chronotype is associated with higher verbal intelligence quotients (Killgore and Killgore, 2007). Conversely, a recent study reported that early chronotype is associated with higher verbal ability after controlling for age and later bedtime (Gibbings et al., 2022). Although some studies, such as those mentioned above, have reported the influence of chronotype on cognitive function in adults, there is a paucity of research on the association of chronotype with cognitive function in children.

Furthermore, there is some evidence regarding the relationship between chronotype and brain structure in adults. One study reported that early chronotype is associated with higher and lower gray matter density in the lateral orbitofrontal cortex and posterior parietal cortex (i.e., precuneus and superior parietal lobule), respectively (Takeuchi et al., 2015). Another study found lower gray matter volume in the lateral occipital cortex and precuneus in adults with early chronotype than in those with late chronotype (Rosenberg et al., 2018). Furthermore, a recent study on adults reported that early chronotype in adults was associated with smaller volume in the right entorhinal cortex (Kim et al., 2023). The various parts of the brain wherein chronotype affects gray matter change seem to be particularly relevant to cognitive function, such as the processing of language (Hickok and Poeppel, 2004; Proverbio and Zani, 2005; He et al., 2013) and memory (Dickerson and Eichenbaum, 2010; Walhovd et al., 2010; Aladro et al., 2018). However, no study to date has identified the association of chronotype with brain structure in children.

This study addresses two research questions. First, is chronotype associated with cognitive function in children? Second, if this is so, is chronotype associated with the brain structures involved in such cognitive function? To answer these questions, we investigated the association between chronotype and cognitive function in a large sample of children from the Adolescent Brain Cognitive Development (ABCD) Study. To further analysis of regional brain volumes, based on the significant association between chronotype and specific cognitive functions, we extracted regions-of-interest (ROI) associated with those functions. Thereafter, we examined the association of chronotype with ROI volumes. We hypothesized that, in children, chronotype would be associated with one or more cognitive functions (i.e., language, memory, and processing speed), and with the regional brain volumes related to these specific cognitive functions. In light of the growing prevalence of sleep-related issues, a better understanding of chronotype may be helpful for considering brain and mental health in childhood.

## 2 Materials and methods

### 2.1 Participants

The ABCD Study is the largest longitudinal study examining child brain development and mental health in the United States (Jernigan and Brown, 2018). Recruitment began in 2016 and ended in 2018; however, the study is ongoing to collect longitudinal data. Full recruitment details of the ABCD Study have been published previously (Garavan et al., 2018). The present study mainly used data from the ABCD 3.0 release, which included 11,878 adolescents aged 9–11 years [Mean age, 9.91 years (Min, 8.91; Max, 11.08)] recruited from 21 data collection sites. All parents provided written informed consent, and all children assented to participate. All procedures complied with the Declaration of Helsinki. The Research Ethics Committee of the University of Fukui approved the data analysis (Assurance No. FU-20210067).

In this analysis, we used the MCTQ data from the 2-year follow-up because there was no baseline data. Accordingly, we obtained data on brain structure and cognitive functioning at the 2-year follow-up. However, demographic data such as handedness, child race/ethnicity, and parental education were obtained at baseline (see Supplementary Table 1 for the variables). First, 5,307 participants who had no data on chronotype were excluded. Second, quality control for structural imaging data and FreeSurfer cortical surface reconstructions were performed manually by the ABCD team. Eight hundred and ninety-three participants who had no T1 quality check and imaging data were excluded. Third, duplicate participants caused by the binding of all data tables were removed (*n* = 357). After the primary data cleaning process (*n* = 5,321), we excluded missing values for chronotype (*n* = 589) and cases ineligible for T1 quality check (*n* = 77), which was extracted using the identifier marked with “0” as unacceptable imaging results by the ABCD team. For quality control of chronotype data in the MCTQ, we excluded children whose reported sleep durations for school days or free days were longer than 15 h or shorter than 3 h per day along with missing values (*n* = 162), because these abnormal sleep durations were more likely caused by mistakes during data collection (Yang et al., 2023). For the final analysis, 4,493 participants were included. Demographic data are shown in Table 1. Data cleaning and statistical analysis were conducted using R (version 4.3.1; The R Foundation for Statistical Computing, Vienna, Austria).

**Table 1:** Demographics.

Characteristic	Total (*n* = 4,493)
Age (months)	143.06 (7.66)
Parental education (years)	15.43 (2.42)
Pubertal status (score)	2.08 (0.62)
Weekly sleep duration (h)	8.83 (1.15)
Chronotype (MSFsc, h)	3.76 (1.52)
Sex (*n*)	
Male	2,451 (54.55)
Female	2,042 (45.47)
Race/ethnicity (*n*)	
White	2,581 (57.47)
Black	493 (10.98)
Hispanic	874 (19.45)
Asian	93 (2.06)
Other	452 (10.06)
Annual household income (US$) (*n*)	
< 49,999	1,120 (24.92)
50,000–74,999	587 (13.07)
75,000–99,999	656 (14.60)
100,000–199,999	1,325 (29.50)
≥ 200,000	483 (10.75)

Data are presented as the mean (SD) or *n* (%). SD, standard deviation; MSFsc, midpoint of sleep on free days, corrected for sleep debt.

### 2.2 Demographic variables and covariates

The following covariates were included as categorical variables and dummy-coded: sex, handedness, race/ethnicity (White, Black, Hispanic, Asian, and other), and medication use. Based on previous studies (Paul et al., 2021; Hamatani et al., 2022; Hiraoka et al., 2023), annual household income was treated as a five-level categorical variable. The following covariates were included as continuous variables: age, parental educational level, pubertal status, weekly sleep duration, and total intracranial volume. Parental educational level was recorded as follows: 12th grade, high school, and general education: 12 years; college and associate degrees: 14 years; bachelor’s degree: 16 years; master’s degree: 18 years; professional and doctoral degrees: 20 years. The pubertal development scale was used to assess pubertal status (Petersen et al., 1988), and completed by both a parent or guardian and the participant, with the two scores averaged for the final value. The abovementioned covariates were selected based on previous ABCD-based studies (Owens et al., 2021; Bernanke et al., 2022; Hamatani et al., 2022; Hiraoka et al., 2023).

### 2.3 Chronotype measures

The MCTQ (Roenneberg et al., 2003) was used to assess chronotype in children. This standardized self-rating scale assesses an individual’s habitual sleep and wake times on school days and free days. The variables consist of (1) sleep start (bedtime and sleep onset latency), (2) sleep end (wake-up time), (3) alarm clock usage, and (4) sleep duration (total amount of time between sleep start and sleep end). Additionally, mid-sleep on free days (MSF) is calculated as the midpoint between sleep onset and wake-up time. Furthermore, MSF needs to be adjusted for sleep debt to obtain the corrected sleep midpoint on free days (MSFsc) because most individuals accumulate sleep debt during the school day and extend their sleep time on free days (Roenneberg et al., 2003). Therefore, MSFsc, also known as chronotype index, is calculated as MSF minus a correction for sleep debt equal to half the difference between sleep duration on free days and average sleep duration over the week, which is only applied if sleep duration on free days is greater than sleep duration on school days. Chronotype was calculated by the ABCD team.

### 2.4 Cognitive measures

Cognitive function (executive function, processing speed, episodic memory, and language) was measured using the NIH Toolbox (Fox et al., 2021; Ott et al., 2022; Nolin et al., 2023). Cognitive tests comprised the flanker inhibitory control and attention task (assessing executive function), pattern comparison processing speed task (assessing processing speed), picture sequence memory task (assessing episodic memory), and picture vocabulary and oral reading recognition tasks (assessing language functioning; see Supplementary materials for the details of each task) (Fox et al., 2021; Ott et al., 2022; Nolin et al., 2023). Age-corrected scores were utilized.

### 2.5 Brain structural measures

Participants were scanned using three 3 T MRI scanners (Siemens, General Electric 750, and Philips) to obtain high-resolution T1-weighted three-dimensional structural images (1 mm isotropic) with acquisition parameters as previously described (Casey et al., 2018). Structural data were preprocessed by the ABCD data team using the standard morphometric pipeline (i.e., skull-stripping, white matter segmentation, etc.) in FreeSurfer (version 5.3.0) (Hagler et al., 2019). First, we extracted 34 regions labeled with the Desikan-Killiany atlas-based classification for cortical regional volume and seven regions labeled with atlas-based segmentation for subcortical regional volumes (68 and 14 regions in total, respectively). Thereafter, based on the significant association between chronotype and certain cognitive measures, we extracted the ROIs associated with these cognitive functions.

### 2.6 Statistical analysis

For all dependent, independent, and continuous variables, outliers were winsorized at 3 standard deviations from the mean (R-package ‘DescTools’). To investigate the association between chronotype and cognitive measures, we adapted a linear mixed-effects model (R-package ‘lmerTest’, ‘MuMIn’, and ‘jtools’) with each cognitive measure modeled as the dependent variable and chronotype as the independent variable. Based on previous studies (Owens et al., 2021; Bernanke et al., 2022; Hamatani et al., 2022; Hiraoka et al., 2023), family ID (used to denote sibling status), multiple data collection sites, and twin or triplet status were modeled as random effects. Covariates included the abovementioned variables. To test the association between chronotype and ROI volumes, we applied a linear mixed-effects model with ROI volumes modeled as the dependent variable and chronotype as the independent variable. In addition to multiple data collection sites and twin or triplet status, we included family ID as a random effect nested inside a random effect of MRI scanner to account for differences across MRI scanners and similarities within families, as previously reported (Heeringa and Berglund, 2020; Owens et al., 2021; Bernanke et al., 2022). Covariates included the abovementioned variables and total intracranial volume. For additional analyses using 82 regional brain volumes, see Supplementary materials. In addition, we adopted a linear mixed-effects model to assess the association between ROI volume and cognitive measures. The ROI volumes were modeled as the independent variable and cognitive measures as the dependent variable, and the covariates were the same variables used to assess the association of chronotype with ROI volumes. The statistical threshold was set at *p* < 0.05, false discovery rate (FDR)-corrected using the Benjamini-Hochberg method. Furthermore, we investigated the mediating effects of chronotype on the relationship between ROI volumes (where chronotype was associated with regional brain volumes) and cognitive measures. For the details of the mediation analysis, see Supplementary materials.

## 3 Results

### 3.1 The association of chronotype with cognitive measures

**Figure 1:**
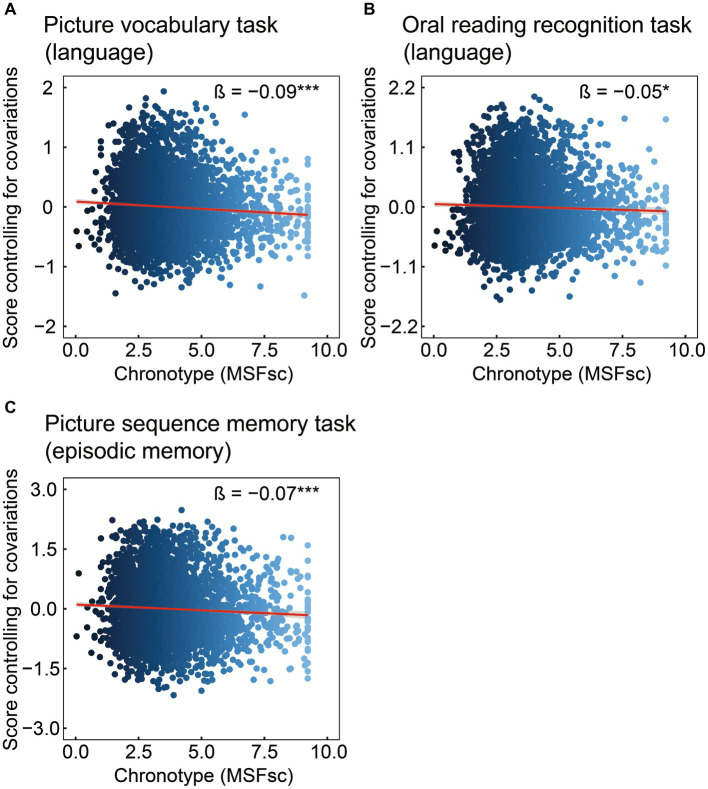
Association of chronotype with cognitive function based on the NIH Toolbox. Chronotype was negatively associated with scores on picture vocabulary **(A)**, oral reading recognition **(B)**, and picture sequence memory **(C)** tasks. Conversely, the associations with scores on the flanker inhibitory control and attention and pattern comparison processing speed tasks were not statistically significant (Table 2). *FDR *p* < 0.05, ***FDR *p* < 0.001. NIH, National Institutes of Health; FDR, false discovery rate; MSFsc, midpoint of sleep on free days, corrected for sleep debt.

Cognitive data are shown in Table 2 and Figure 1. Chronotype was negatively associated with scores on the picture vocabulary (Figure 1A: FDR *p* < 0.001), oral reading recognition (Figure 1B: FDR *p* = 0.014), and picture sequence memory (Figure 1C: FDR *p* < 0.001) tasks, indicating that late chronotype is associated with lower levels of language and episodic memory.

**Table 2:** Cognitive characteristic associated with chronotype.

NIH toolbox	Standardized coefficients (*β*)	95% CI	*R* ^2^	*t*	FDR-*P*
FICA	−0.02	−0.05, 0.02	0.04	0.96	0.338
PV	−0.09	−0.12, −0.05	0.23	5.16	< 0.001
ORR	−0.05	−0.08, −0.01	0.12	2.64	0.014
PCPS	−0.03	−0.07, 0.003	0.05	1.81	0.087
PSM	−0.07	−0.11, −0.04	0.10	4.06	< 0.001

NIH, National Institutes of Health; FICA, flanker inhibitory control and attention; PV, picture vocabulary; ORR, oral reading recognition; PCPS, pattern comparison processing speed; PSM, picture sequence memory; CI, confidence interval; FDR, false discovery rate.

### 3.2 The association of chronotype with brain structure

**Figure 2:**
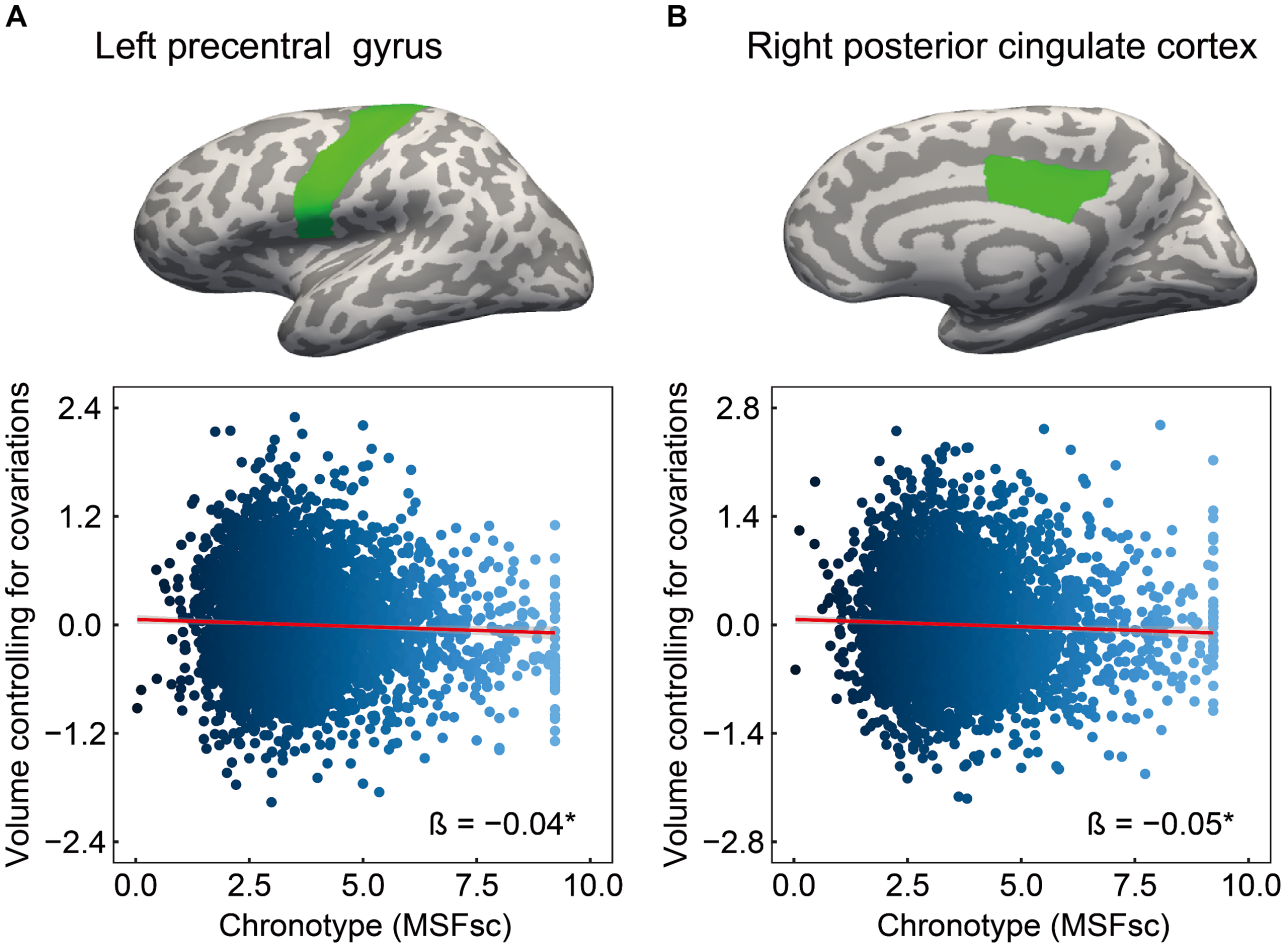
Association of chronotype with cognition-related brain structure. Considering ROIs in both hemispheres that are associated with language and episodic memory (as chronotype was found to be associated with scores on picture vocabulary, oral reading recognition, and picture sequence memory tasks), chronotype showed negative associations with volumes in the left precentral **(A)** and right posterior cingulate **(B)**. In other regions, the associations were not significant (Supplementary Table 3). *FDR *p* < 0.05. ROI, region of interest; FDR, false discovery rate; MSFsc, midpoint of sleep on free days, corrected for sleep debt.

The cognitive results revealed associations of chronotype with language and episodic memory functions. Regarding the neural basis of language, previous studies have proposed an anatomical topographic organization such that speech perception is represented in the superior temporal gyrus, sound-meaning processing is associated with the middle temporal and inferior temporal gyri, the auditory-motor interface is involved in the supramarginal gyrus and superior parietal cortex, and articulatory-based speech codes are indicated in the inferior frontal gyrus and primary motor area (Hickok and Poeppel, 2004; Proverbio and Zani, 2005; Cattaneo, 2013). In addition, the volumes of the superior parietal cortex, supramarginal gyrus, superior frontal gyrus, and lateral occipital cortex are associated with performance in phonological decoding tasks (He et al., 2013). Based on these findings, we extracted 24 ROIs as language-related areas (see Supplementary Table 2 for details on these brain regions). On investigating the association between chronotype and these ROI volumes, chronotype was found to be negatively associated with volume in the left precentral gyrus (Table 3 and Figure 2A: FDR *p* = 0.049).

**Table 3:** Brain structural characteristics associated with chronotype.

Brain region	Standardized coefficient (*β*)	95% CI	*R* ^2^	*t*	FDR-*P*
Language-related area					
L precentral	−0.04	−0.07, −0.01	0.49	3.01	0.049
Episodic memory-related area					
R posterior cingulate	−0.05	−0.08, −0.02	0.31	3.04	0.049

CI, confidence interval; FDR, false discovery rate; L, left; R, right.

Regarding the neural basis of memory, besides the middle temporal lobe (especially in the hippocampus, entorhinal area, and parahippocampal gyrus) (Dickerson and Eichenbaum, 2010; Walhovd et al., 2010; Aladro et al., 2018), the posterior cingulate cortex, precuneus, inferior parietal cortex, and lateral orbitofrontal cortex are thought to influence episodic memory processing (Cavanna and Trimble, 2006; Dickerson and Eichenbaum, 2010; Walhovd et al., 2010; Leech and Sharp, 2014; Aladro et al., 2018). Among the various brain regions associated with the memory system, structural changes in the entorhinal cortex, precuneus, and posterior cingulate cortex have been associated with performance in episodic memory tasks (Walhovd et al., 2010; Özyurt et al., 2017; Pelletier et al., 2017; Aladro et al., 2018). Based on these findings, we extracted 14 ROIs as episodic memory-related areas (see Supplementary Table 2 for details on these brain regions). Investigating the association between chronotype and these ROI volumes, we found that chronotype was negatively associated with the right posterior cingulate cortex volume (Table 3 and Figure 2B: FDR *p* = 0.049). These results show that late chronotype is associated with smaller volumes of the left precentral gyrus and right posterior cingulate cortex. For significant associations between chronotype and the 82 regional brain volumes, see Supplementary results and Supplementary Table 4.

**Figure 3:**
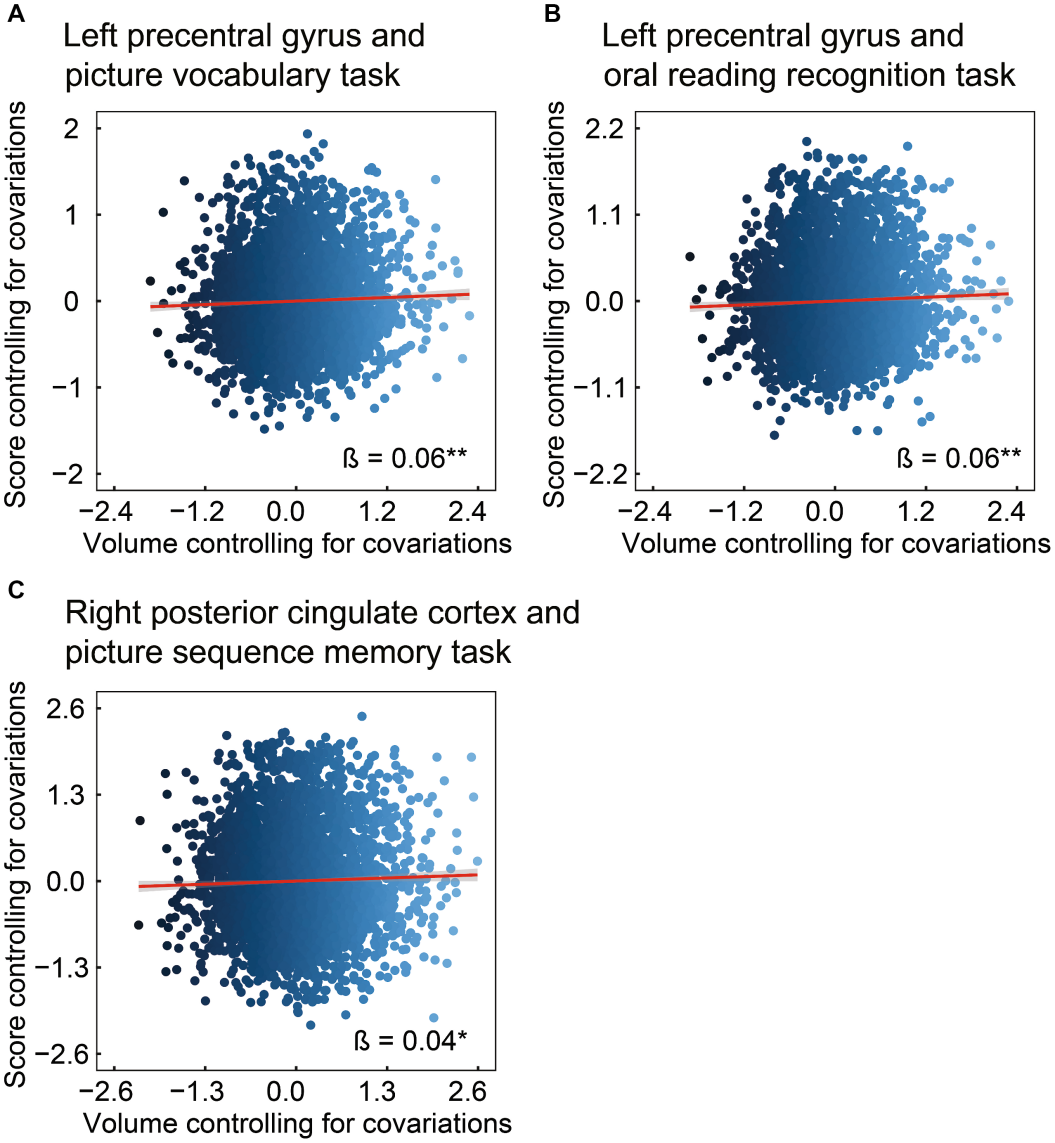
Association of brain structure with language or episodic memory. Considering ROIs in the left precentral and right posterior cingulate (i.e., regional volumes that were found to be associated with chronotype), the volume in the left precentral was positively associated with the scores on the picture vocabulary **(A)** and oral reading recognition **(B)** tasks. In addition, the volume of the posterior cingulate was positively associated with the scores on the picture sequence memory task **(C)**. *FDR *p* < 0.05, **FDR *p* < 0.01. ROI, region of interest; FDR, false discovery rate.

Subsequently, we investigated the associations between the volumes of ROIs in the left precentral gyrus and right posterior cingulate cortex, and scores on the picture vocabulary, oral reading recognition, and picture sequence memory tasks (Figure 3). The left precentral gyrus was positively associated with scores on the picture vocabulary (Figure 3A: *β* = 0.06, 95% CI [0.02, 0.09], *R*^2^ = 0.23, *t* = 2.83, FDR *p* = 0.007) and oral reading recognition (Figure 3B: *β* = 0.06, 95% CI [0.02, 0.10], *R*^2^ = 0.13, *t* = 2.90. FDR *p* = 0.007) tasks. In addition, the right posterior cingulate cortex was positively associated with scores on the picture sequence memory task (Figure 3C: *β* = 0.04, 95% CI [0.004, 0.08], *R*^2^ = 0.10, *t* = 2.17, FDR *p* = 0.030). These results indicate that larger volumes of the precentral gyrus and posterior cingulate cortex are associated with higher levels of language and episodic memory, respectively. For the mediating effect of chronotype on the relationship between these regional volumes and language and episodic memory performances, see Supplementary results and Supplementary Figure 1.

## 4 Discussion

This study investigated the relationship between chronotype and cognitive function in children. Subsequently, the relationship between chronotype and specific regional brain volumes related to cognitive function was examined. Chronotype is negatively associated with the scores on picture vocabulary, oral reading recognition, and picture sequence memory tasks (Figure 1). In addition, chronotype is negatively associated with the volumes of the left precentral gyrus and right posterior cingulate cortex (Figure 2). These findings suggest that late chronotype is associated not only with low language and episodic memory performance but also with reduced volumes of the precentral gyrus and posterior cingulate cortex. Furthermore, we examined the relationship between these regional brain volumes, and language and episodic memory performance, finding that the precentral gyrus and posterior cingulate cortex are positively associated with language and episodic memory skills, respectively (Figure 3). These findings may suggest that children with late chronotype have lower language comprehension and episodic memory and smaller brain volumes in the left precentral gyrus and right posterior cingulate cortex associated with these cognitive functions.

In contrast to previous reports that late chronotype in adults is associated with better memory and verbal ability (Roberts and Kyllonen, 1999; Killgore and Killgore, 2007; Gorgol et al., 2020), this study revealed a negative association between chronotype, and picture vocabulary, oral reading recognition, and picture sequence memory task performances, suggesting that late chronotype is associated with lower vocabulary, reading, and episodic memory skills in children. A possible reason for this discrepancy is age-related differences in preferred chronotype. Roenneberg et al. (2004) found that children (aged 10–12 years) exhibit an early chronotype and tend to shift toward a late chronotype around the age of 20 years, suggesting that early chronotype may be biologically preferred during childhood (Roenneberg et al., 2004; Randler, 2011). Thus, in children with a mean age of 11.09 years, as in the current study, early chronotype may be preferable to maintain cognitive performance. Furthermore, late chronotype is associated with larger daily sleep debt, morning sleepiness, and poorer sleep quality (Taillard et al., 1999, 2004; Khan et al., 2020). As good night-time sleep has been implicated in better language and memory consolidation (Cousins et al., 2019; Berens et al., 2022), children with late chronotype may be particularly vulnerable to impaired vocabulary, reading, and episodic memory skills, possibly due to accumulated sleep debt.

Based on previous findings (Hickok and Poeppel, 2004; Proverbio and Zani, 2005; Cavanna and Trimble, 2006; Dickerson and Eichenbaum, 2010; Walhovd et al., 2010; Cattaneo, 2013; Leech and Sharp, 2014; Aladro et al., 2018) and our behavioral results, language-related and episodic memory-related ROI analysis revealed negative associations between chronotype, and the left precentral gyrus and right posterior cingulate cortex volumes. Moreover, a larger left precentral gyrus volume was associated with higher scores on both the picture vocabulary and oral reading recognition tasks. Additionally, greater volume in the right posterior cingulate cortex was associated with better performance on the picture sequence memory task. These findings suggest that in children, late chronotype is associated with smaller volumes in the left precentral gyrus and right posterior cingulate cortex involved in language and episodic memory skills. In contrast, a few studies have reported that early chronotypes were associated with smaller gray matter volume in the entorhinal cortex, posterior parietal cortex, lateral occipital cortex, and precuneus in adults (Takeuchi et al., 2015; Rosenberg et al., 2018; Kim et al., 2023). Because these studies considered adults who may have gradually become late chronotypes post-childhood (Roenneberg et al., 2004), the relationship between the previous findings and our results in children cannot be clearly interpreted. Although sleep duration may be associated with structural brain health (Tai et al., 2022), the current results highlight the impact of late chronotype in children on the structural deterioration of the precentral gyrus and posterior cingulate cortex without poor sleep duration.

The precentral gyrus is involved in motor control (Rizzolatti and Luppino, 2001) and language processing (Hickok and Poeppel, 2004; Lee et al., 2014). Schug et al. (2022) reported that bilingual children have larger gray matter volume in the left precentral gyrus compared to monolingual children, suggesting that such structural characteristics play an important role in speech motor control (Behroozmand et al., 2015) and its feedback processing (Parkinson et al., 2012), which is required for precise vocabulary knowledge and speech production. This region is also associated with sleep deprivation (Huang et al., 2022), and is engaged in a premediated state to prepare the brain for motor execution and coordination (Chenji et al., 2016). In our study, a larger left precentral gyrus volume was associated with higher vocabulary knowledge and reading skills. Furthermore, the left precentral gyrus volume has a significant direct effect on the vocabulary knowledge level. Moreover, such volume has a significant indirect effect on the vocabulary knowledge level that is partially mediated through chronotype (Supplementary Figure 1A). While precentral gyrus volume was associated with the maintenance of vocabulary knowledge, these mechanisms may be partially mediated by chronotype.

In addition, the posterior cingulate cortex, a key node in the default mode network (Fransson and Marrelec, 2008), is involved in planning for the future, internal/external thought, attention, and episodic memory (i.e., autobiographical memories) (Hahn et al., 2007; Dickerson and Eichenbaum, 2010; Leech and Sharp, 2014), suggesting that structural and functional anomalies in this region are associated with the suppression of self-referential processing (Dastjerdi et al., 2011). Moreover, a recent study reported that changes in functional connectivity in the posterior cingulate cortex seed predict sleepiness (Facer-Childs et al., 2019), suggesting that abnormalities in the neural network in the posterior cingulate cortex may influence the induction of sleepiness. Some studies reported that poorer sleep quality and sleep abnormality are associated with a reduction in the volume of the posterior cingulate cortex (Heidbreder et al., 2017; Liu et al., 2022). As late chronotype has been implicated in morning sleepiness and poorer sleep quality (Taillard et al., 1999, 2004; Khan et al., 2020), the daily accumulation of late chronotype-related sleep debt may strongly influence structural anomalies in the posterior cingulate cortex. Similarly, a larger posterior cingulate cortex volume was associated with better performance in episodic memory tasks, suggesting its involvement in memory maintenance. Furthermore, although the right posterior cingulate cortex volume has no significant direct effect on episodic memory level, such volume has a significant indirect effect on episodic memory level that is fully mediated through chronotype (Supplementary Figure 1C). This suggests that the posterior cingulate cortex volume may not be directly related to episodic memory performance. Alternatively, this structure may be associated with the maintenance of episodic memory through chronotype.

Our study has several limitations. First, our design was cross-sectional; thus, we plan to investigate longitudinally whether late chronotype is associated with behavioral changes and brain structural development in childhood. Second, this study used a restricted cognitive assessment battery from the NIH Toolbox because of missing data on working memory-related tasks. Therefore, future research should examine associations with late chronotype using a broader spectrum of neuropsychological measures in childhood. Finally, in our design, the ROI volumes selected based on chronotype-related cognitive characteristics were extracted from 24 ROIs as language-related areas and 14 ROIs as episodic memory-related areas, as previously reported (see Results section and Supplementary Table 2). Although previous studies have indicated that ROI extraction related to specific cognitive functions is effective in understanding the neural basis of cognitive function (Kanwisher et al., 1997; D’Esposito et al., 1999; Banich et al., 2009; Kurth et al., 2010; Matyi and Spielberg, 2022), the limitation may include the selection of speculative ROIs. Therefore, we investigated linear mixed-effect models for associations between chronotype and 82 regional brain volumes. Our uncorrected results (*p* < 0.05) suggest that late chronotype was associated with smaller volumes in the left precentral gyrus, left lateral orbitofrontal cortex, right posterior cingulate cortex, right rostral middle frontal cortex, right pars orbitalis, and right superior parietal cortex, with a larger volume in the right cuneus (Supplementary Table 4). However, such additional analyses should be interpreted with caution because these uncorrected results were not significant after FDR correction.

## 5 Conclusion

For the first time, associations were found between chronotype, and behavioral and brain structural characteristics in childhood. Behaviorally, late chronotype was associated with lower levels of vocabulary, reading, and episodic memory. Structurally, late chronotype was associated with volume reduction in the language-related left precentral gyrus and episodic memory-related right posterior cingulate cortex. Our results suggest that children with late chronotype have lower language comprehension and episodic memory and smaller brain volumes in the left precentral gyrus and right posterior cingulate cortex associated with these cognitive functions.

## Data availability statement

Publicly available datasets were analyzed in this study. This data can be found here: data can be accessed by registering with the ABCD Study at https://nda.nih.gov/abcd. Information on how to access ABCD data through the NDA is available on the ABCD Study data-sharing webpage: https://abcdstudy.org/scientists_data_sharing.html. Instructions on how to create an NDA study are available at https://nda.nih.gov/nda/webinars-and-tutorials. R codes for this analysis are available at https://osf.io/ht43q after acceptance.

## Ethics statement

The studies involving humans were approved by The Research Ethics Committee of the University of Fukui. The studies were conducted in accordance with the local legislation and institutional requirements. Written informed consent for participation in this study was provided by the participants’ legal guardians/next of kin.

## Author contributions

MY: Writing – original draft, Writing – review & editing, Conceptualization, Data curation, Formal analysis, Funding acquisition, Investigation, Methodology, Project administration, Resources, Software, Validation, Visualization. QS: Methodology, Validation, Writing – review & editing. YM: Conceptualization, Funding acquisition, Methodology, Project administration, Resources, Supervision, Writing – review & editing.
