# Supplementary material for: Association of chronotype with language and episodic memory processing in children: implications for brain structure
Source: Front Integr Neurosci. 2024 Aug 7;18:1437585. doi: 10.3389/fnint.2024.1437585 (PMC11335642; doi:10.3389/fnint.2024.1437585)
Supplement: Supplementary file 1 [file Data_Sheet_1.docx]

Supplementary Material

# Supplementary Methods

**National Institutes of Health Toolbox tasks**

The National Institutes of Health Toolbox Tasks were conducted using an iPad-based program in the ABCD Study (Luciana et al., 2018). In the flanker inhibitory control and attention task, participants were instructed to indicate the left-right orientation of a central arrow flanked by two arrows pointing in the same (congruent) or different (incongruent) direction. Twenty trials were presented, with the flanking arrows alternating randomly between congruence and incongruence with the middle arrow. The score was generated by combining response time and accuracy vectors. In the pattern comparison processing speed task, participants were instructed to use their dominant hand to tap “yes” if the stimuli presented on the screen were the same and “no” if they were not. Stimuli were presented in succession over a period of 90 s, with the participant responding to as many stimuli as possible within this period. The score was based on the number of items (out of a possible 130) answered correctly in the timeframe. In the picture sequence memory task, images and verbal statements of events (e.g., “going to the park”) were presented sequentially and assigned corresponding positions on the screen. Subsequently, images were presented scrambled in the screen center and participants were instructed to replicate the sequence by dragging the images to their appropriate positions. Sequences varied from 6–18 pictures, based on the participant’s chronological age. The score was generated based on the number of adjacent image pairs placed in the correct order for each trial. In the picture vocabulary task, participants were instructed to select an image from a set of four that corresponded to a read-out word. Twenty-five trials were represented, and the scores were based on the number of correct responses. In the oral reading recognition task, participants were instructed to read out words that were presented on the screen. The participants received one of the four forms (form 1, 70 items; form 2, 101 items; form 3, 120 items; form 4, 125 items) and attempted items until they either completed the prescribed number of items for their form or mispronounced 10 words in a row. The scores were based on the number of correct responses.

**Linear mixed-effect model for associations between chronotype and 82 regional brain volumes**

We investigated additional linear mixed-effect models for associations between chronotype and 82 regional brain volumes. A linear mixed-effect model was used with each regional brain volume as the dependent variable and chronotype as the independent variable. In addition to multiple data collection sites and twin or triplet status, we included family ID (sibling status) as a random effect nested inside a random effect of the magnetic resonance imaging scanner to account for the large number of siblings and multiple data collection sites, as previously recommended (Bernanke et al., 2022; Heeringa and Berglund, 2020; Owens et al., 2021). For covariates, see “Demographic Variables and Covariates” in the “Materials and Methods” section of the main text. The statistical threshold was set at *P* < 0.05, false discovery rate (FDR)-corrected using the Benjamini–Hochberg method.

**Mediation analysis**

Based on significant associations between chronotype and cognitive function (language and episodic memory) and regions-of-interest (ROI) volumes (left precentral gyrus and right posterior cingulate cortex, see “Results” section in the main text), we investigated the mediating effect of chronotype on the relationship of ROI volumes with language and episodic memory performances. First, the left precentral gyrus volume and score on the picture vocabulary task were residualized for random effect and covariates using the linear mixed-effect model. These measures were then converted to z-scores. Further, we conducted a standard three-variable mediation analysis (R-package ‘lavaan’) to estimate the significance of the mediating effect by using the bias-corrected bootstrap approach (with 10,000 random samplings). Second, the left precentral gyrus volume and score on the oral reading recognition task were residualized for random effect and covariates using the linear mixed-effect model, and these measures were converted to z-scores. This standard three-variable mediation analysis was performed using the abovementioned method. Finally, the right posterior cingulate cortex volume and scores on the picture sequence memory task were residualized for random effect and covariates using the linear mixed-effect model, and the measures were also converted to z-scores. Similarly, this standard three-variable mediation analysis was performed using the aforementioned method.

# Supplementary Results

**The association between chronotype and 82 regional brain volumes**

Based on FDR-corrected thresholds (*P* < 0.05), there were no significant associations between chronotype and 82 regional brain volumes (**Supplementary Table 4**). However, uncorrected results showed that chronotype was negatively associated with volumes in the left precentral gyrus (uncorrected *P* = 0.003; FDR *P* = 0.108), left lateral orbitofrontal cortex (uncorrected *P* = 0.029; FDR *P* = 0.577), right posterior cingulate cortex (uncorrected *P* = 0.002; FDR *P* = 0.108), right rostral middle frontal cortex (uncorrected *P* = 0.016; FDR *P* = 0.425), right pars orbitalis (uncorrected *P* = 0.026; FDR *P* = 0.480), and right superior parietal cortex (uncorrected *P* = 0.047; FDR *P* = 0.577). Additionally, chronotype was positively associated with the right cuneus volume (uncorrected *P* = 0.049; FDR *P* = 0.577). These uncorrected results suggest that late chronotype may be associated with these regional brain volume reductions or enlargements.

**The mediating effect of chronotype on the relationship between ROI volumes and language and episodic memory performances**

We investigated the mediating effects of chronotype on the relationship between ROI volumes (where chronotype was associated with precentral gyrus and posterior cingulate cortex volumes, as illustrated in **Figure 2**) and language and episodic memory measures. First, the left precentral gyrus volume had a significant direct effect on the picture vocabulary task scores (*β* = 0.04, 95% CI [0.004, 0.07], *P* = 0.025). Moreover, such volume had a significant indirect effect on the picture vocabulary task scores that are transmitted through chronotype (**Supplementary Figure 1A**: *β* = 0.003, 95% CI [0.001, 0.006], *P* = 0.021). In contrast, there was no significant mediating effect of chronotype on the relationship between such volume and the oral reading recognition task scores (**Supplementary Figure 1B**: *β* = 0.002, 95% CI [0.0001, 0.004], *P* = 0.095). Moreover, although the right posterior cingulate cortex volume had no significant direct effect on the picture sequence memory task scores (*β* = 0.03, 95% CI [-0.002, 0.06], *P* = 0.068), such volume had a significant indirect effect on the picture sequence memory task scores that is transmitted through chronotype (Supplementary Figure 1C: *β* = 0.002, 95% CI [0.0004, 0.005], *P* = 0.048).

# Supplementary Tables and Figures

## Supplementary Tables

**Supplementary Table 1.** Variables used in this study

| Variable | Data file |
| --- | --- |
| NIH Toolbox: flanker inhibitory control and attention | abcd_tbss01 |
| NIH Toolbox: pattern comparison processing speed |  |
| NIH Toolbox: picture sequence memory |  |
| NIH Toolbox: picture vocabulary |  |
| NIH Toolbox: oral reading recognition |  |
| Gray matter volume for 34 cortical regions (Desikan atlas-based classification; 68 regions in total) | abcd_smrip101 |
| Gray matter volume for 7 subcortical regions (atlas-based classification; 14 regions in total), Intracranial volume | abcd_smrip201 |
| Handedness | abcd_ehis01 |
| Chronotype, weekly sleep duration | abcd_mcqc01 |
| FreeSurfer quality control | abcd_imgincl01 |
| Site ID | abcd_lt01 |
| MRI scanner number | abcd_mri01 |
| Medication use | medsy01 |
| Race/ethnicity, sibling status, twin or triplet status | acspsw03 |
| Home environment (education and income) | pdem02 |
| Pubertal status | abcd_ppdms01 (parent/guardian)  abcd_ypdms01(participants) |

MRI, magnetic resonance imaging.

**Supplementary Table 2.** Summary of language-related and episodic memory-related area

| Brain region |
| --- |
| Language-related areas (bilateral 24 regions) |
| Caudal middle frontal, Fusiform, Precentral, Inferior temporal, Middle temporal, Lateral occipital, Rostral middle frontal, Superior parietal, Superior temporal, Superior frontal, Supramarginal, Insula |
|  |
| Episodic memory-related areas (bilateral 14 regions) |
| Entorhinal, Parahippocampal, Posterior cingulate, Precuneus, inferior parietal, lateral orbitofrontal, Hippocampus |

**Supplementary Table 3.** Brain structural characteristics associated with chronotype

| Brain region | Standardized coefficient (*β*) | 95% CI | *R*^2^ | *t* | FDR-*P* |
| --- | --- | --- | --- | --- | --- |
| **Language-related area** | | | | | |
| L caudal middle frontal | -0.009 | -0.04, 0.02 | 0.30 | 0.59 | 0.810 |
| R caudal middle frontal | -0.001 | -0.03, 0.03 | 0.30 | 0.06 | 0.975 |
| L fusiform | -0.02 | -0.05, 0.006 | 0.38 | 1.51 | 0.446 |
| R fusiform | -0.01 | -0.04, 0.02 | 0.47 | 0.79 | 0.775 |
| L inferior temporal | -0.01 | -0.04, 0.02 | 0.42 | 0.77 | 0.775 |
| R inferior temporal | -0.002 | -0.03, 0.03 | 0.43 | 0.10 | 0.975 |
| L lateral occipital | -0.0009 | -0.03, 0.03 | 0.43 | 0.06 | 0.975 |
| R lateral occipital | -0.01 | -0.04, 0.02 | 0.45 | 0.81 | 0.775 |
| L middle temporal | -0.00002 | -0.03, 0.03 | 0.44 | 0.002 | 0.999 |
| R middle temporal | -0.01 | -0.04, 0.01 | 0.51 | 0.84 | 0.775 |
| R precentral | -0.02 | -0.04, 0.01 | 0.46 | 1.14 | 0.611 |
| L rostral middle frontal | -0.02 | -0.05, 0.006 | 0.49 | 1.53 | 0.446 |
| R rostral middle frontal | -0.03 | -0.06, -0.006 | 0.45 | 2.42 | 0.197 |
| L superior frontal | -0.02 | -0.05, 0.004 | 0.53 | 1.63 | 0.446 |
| R superior frontal | -0.01 | -0.04, 0.01 | 0.50 | 1.00 | 0.710 |
| L superior parietal | -0.02 | -0.05, -0.005 | 0.40 | 1.62 | 0.446 |
| R superior parietal | -0.03 | -0.06, -0.0004 | 0.41 | 1.99 | 0.355 |
| L superior temporal | -0.02 | -0.05, 0.003 | 0.48 | 1.74 | 0.446 |
| R superior temporal | 0.008 | -0.02, 0.04 | 0.46 | 0.61 | 0.810 |
| L supramarginal | -0.004 | -0.02, 0.03 | 0.46 | 0.30 | 0.958 |
| R supramarginal | -0.02 | -0.05, 0.01 | 0.38 | 1.13 | 0.611 |
| L insula | 0.005 | -0.02, 0.03 | 0.48 | 0.35 | 0.958 |
| R insula | -0.003 | -0.03, 0.02 | 0.49 | 0.28 | 0.958 |
| **Episodic memory-related area** | | | | | |
| L entorhinal | -0.01 | -0.04, 0.02 | 0.20 | 0.67 | 0.810 |
| R entorhinal | -0.02 | -0.06, 0.01 | 0.16 | 1.31 | 0.556 |
| L lateral orbitofrontal | -0.03 | -0.06, -0.003 | 0.50 | 2.18 | 0.278 |
| R lateral orbitofrontal | -0.02 | -0.05, 0.007 | 0.38 | 1.47 | 0.446 |
| L inferior parietal | -0.002 | -0.03, 0.03 | 0.38 | 0.15 | 0.975 |
| R inferior parietal | -0.002 | -0.03, 0.03 | 0.44 | 0.16 | 0.975 |
| L parahippocampal | -0.02 | -0.05, 0.01 | 0.26 | 1.26 | 0.565 |
| R parahippocampal | -0.03 | -0.06, 0.006 | 0.32 | 1.60 | 0.446 |
| L posterior cingulate | -0.005 | -0.04, 0.03 | 0.32 | 0.33 | 0.958 |
| L precuneus | -0.008 | -0.03, 0.02 | 0.49 | 0.60 | 0.810 |
| R precuneus | -0.001 | -0.03, 0.02 | 0.51 | 0.10 | 0.975 |
| L hippocampus | -0.008 | -0.04, 0.02 | 0.36 | 0.55 | 0.815 |
| R hippocampus | -0.01 | -0.04, 0.02 | 0.41 | 0.76 | 0.775 |

CI, confidence interval; FDR, false discovery rate; L, left; R, right.

**Supplementary Table 4.** Associations between chronotype and 82 regional brain volumes

| Brain region | *β* | 95% CI | *R*^2^ | *t* | Uncorrected-*P* | FDR-*P* |
| --- | --- | --- | --- | --- | --- | --- |
| **Left** | | | | | | |
| Precentral | -0.04 | -0.07, -0.01 | 0.49 | 3.01 | 0.003 | 0.108 |
| Lateral orbitofrontal | -0.03 | -0.06, -0.0004 | 0.50 | 2.18 | 0.029 | 0.577 |
| Pars orbitalis | -0.03 | -0.06, 0.002 | 0.31 | 1.83 | 0.067 | 0.577 |
| Superior tempotral | -0.02 | -0.05, 0.003 | 0.48 | 1.74 | 0.081 | 0.577 |
| Superior frontal | -0.03 | -0.06, 0.004 | 0.53 | 1.63 | 0.103 | 0.577 |
| Caudate | -0.03 | -0.06, 0.005 | 0.32 | 1.63 | 0.103 | 0.577 |
| Superior parietal | -0.02 | -0.05, 0.005 | 0.40 | 1.62 | 0.105 | 0.577 |
| Rostral middle frontal | -0.02 | -0.05, 0.006 | 0.49 | 1.53 | 0.125 | 0.577 |
| fusiform | -0.02 | -0.05, 0.007 | 0.38 | 1.51 | 0.130 | 0.577 |
| Thalamus proper | -0.02 | -0.04, 0.005 | 0.61 | 1.51 | 0.132 | 0.577 |
| Caudal anterior cingulate | -0.03 | -0.06, 0.01 | 0.18 | 1.45 | 0.147 | 0.577 |
| Isthmus cingulate | -0.02 | -0.05, 0.01 | 0.36 | 1.29 | 0.197 | 0.645 |
| Parahippocampal | -0.02 | -0.05, 0.01 | 0.26 | 1.26 | 0.208 | 0.656 |
| Putamen | 0.02 | -0.01, 0.05 | 0.31 | 1.11 | 0.265 | 0.725 |
| Banks of superior temporal sulcus | -0.02 | -0.05, 0.02 | 0.24 | 1.02 | 0.309 | 0.789 |
| Lingual | -0.02 | -0.05, 0.02 | 0.28 | 0.96 | 0.338 | 0.810 |
| Cuneus | 0.01 | -0.02, 0.04 | 0.25 | 0.80 | 0.423 | 0.810 |
| Accumbens-area | 0.01 | -0.02, 0.04 | 0.21 | 0.79 | 0.428 | 0.810 |
| Rostral anterior cingulate | -0.01 | -0.04, 0.02 | 0.36 | 0.79 | 0.430 | 0.810 |
| Inferior temporal | -0.01 | -0.04, 0.02 | 0.42 | 0.77 | 0.441 | 0.810 |
| Temporal pole | -0.01 | -0.05, 0.02 | 0.13 | 0.70 | 0.484 | 0.810 |
| Entorhinal | -0.01 | -0.04, 0.02 | 0.20 | 0.67 | 0.503 | 0.816 |
| Transverse temporal | -0.01 | -0.04, 0.02 | 0.23 | 0.65 | 0.517 | 0.816 |
| Precuneus | -0.008 | -0.03, 0.02 | 0.49 | 0.60 | 0.546 | 0.816 |
| Caudal middle frontal | -0.009 | -0.04, 0.02 | 0.30 | 0.59 | 0.554 | 0.816 |
| Postcentral | -0.009 | -0.04, 0.02 | 0.40 | 0.59 | 0.555 | 0.816 |
| Pallidum | 0.01 | -0.02, 0.04 | 0.26 | 0.59 | 0.558 | 0.816 |
| Hippocampus | -0.008 | -0.04, 0.02 | 0.36 | 0.55 | 0.579 | 0.833 |
| Insula | 0.005 | -0.02, 0.03 | 0.48 | 0.35 | 0.724 | 0.943 |
| Medial orbitofrontal | -0.005 | -0.03, 0.02 | 0.29 | 0.34 | 0.736 | 0.943 |
| Posterior cingulate | -0.005 | -0.04, 0.03 | 0.32 | 0.33 | 0.738 | 0.943 |
| Pars triangularis | -0.006 | -0.04, 0.03 | 0.18 | 0.32 | 0.746 | 0.943 |
| Pars opercularis | -0.005 | -0.04, 0.03 | 0.22 | 0.32 | 0.751 | 0.943 |
| Frontal pole | -0.005 | -0.04, 0.03 | 0.14 | 0.31 | 0.755 | 0.943 |
| Supramarginal | 0.004 | -0.02, 0.03 | 0.46 | 0.30 | 0.762 | 0.943 |
| Pericalcarine | 0.003 | -0.03, 0.04 | 0.22 | 0.21 | 0.834 | 0.957 |
| Inferior parietal | 0.002 | -0.03, 0.03 | 0.38 | 0.15 | 0.883 | 0.961 |
| Paracentral | 0.002 | -0.03, 0.03 | 0.25 | 0.14 | 0.886 | 0.961 |
| Amygdala | -0.0009 | -0.03, 0.03 | 0.33 | 0.07 | 0.947 | 0.961 |
| Lateral occipital | -0.0009 | -0.03, 0.03 | 0.43 | 0.06 | 0.949 | 0.961 |
| Middle temporal | -0.00002 | -0.03, 0.03 | 0.44 | 0.001 | 0.999 | 0.999 |
| **Right** | | | | | | |
| Posterior cingulate | -0.05 | -0.08, -0.02 | 0.31 | 3.04 | 0.002 | 0.108 |
| Rostral middle frontal | -0.03 | -0.06, -0.006 | 0.45 | 2.42 | 0.016 | 0.425 |
| Pars orbitalis | -0.04 | -0.07, -0.004 | 0.26 | 2.23 | 0.026 | 0.480 |
| Superior parietal | -0.03 | -0.06, -0.0004 | 0.41 | 1.99 | 0.047 | 0.577 |
| Cuneus | 0.03 | 1.65, 0.06 | 0.28 | 1.96 | 0.049 | 0.577 |
| Pars triangularis | -0.03 | -0.07, 0.001 | 0.16 | 1.90 | 0.057 | 0.577 |
| Parahippocampal | -0.03 | -0.06, 0.006 | 0.32 | 1.60 | 0.109 | 0.577 |
| Isthmus cingulate | -0.02 | -0.06, 0.007 | 0.29 | 1.54 | 0.123 | 0.577 |
| Lateral orbitofrontal | -0.02 | -0.05, 0.007 | 0.38 | 1.47 | 0.141 | 0.577 |
| Caudal anterior cingulate | -0.03 | -0.06. 0.009 | 0.16 | 1.45 | 0.148 | 0.577 |
| Pars opercularis | -0.02 | -0.06, 0.009 | 0.23 | 1.42 | 0.155 | 0.577 |
| Caudate | -0.02 | -0.05, 0.009 | 0.33 | 1.40 | 0.163 | 0.580 |
| Entorhinal | -0.02 | -0.06. 0.01 | 0.16 | 1.31 | 0.190 | 0.645 |
| Thalamus proper | -0.01 | -0.04, 0.01 | 0.55 | 1.15 | 0.249 | 0.725 |
| Precentral | -0.02 | -0.04, 0.01 | 0.46 | 1.14 | 0.255 | 0.725 |
| Supramarginal | -0.02 | -0.05, 0.01 | 0.38 | 1.13 | 0.257 | 0.725 |
| Lingual | 0.02 | -0.01. 0.05 | 0.31 | 1.11 | 0.265 | 0.725 |
| Superior frontal | -0.01 | -0.04, 0.01 | 0.50 | 1.00 | 0.317 | 0.789 |
| Paracentral | -0.01 | -0.05, 0.02 | 0.25 | 0.94 | 0.348 | 0.810 |
| Amygdala | -0.01 | -0.04, 0.02 | 0.34 | 0.88 | 0.377 | 0.810 |
| Middle temporal | -0.01 | -0.04, 0.01 | 0.51 | 0.84 | 0.401 | 0.810 |
| Lateral occipital | -0.01 | -0.04, 0.02 | 0.45 | 0.82 | 0.414 | 0.810 |
| Fusiform | -0.01 | -0.04, 0.02 | 0.47 | 0.79 | 0.432 | 0.810 |
| Hippocampus | -0.01 | -0.04, 0.02 | 0.41 | 0.76 | 0.449 | 0.810 |
| Transverse temporal | -0.01 | -0.05, 0.02 | 0.23 | 0.74 | 0.461 | 0.810 |
| Postcentral | -0.01 | -0.04, 0.02 | 0.36 | 0.73 | 0.464 | 0.810 |
| Temporal pole | -0.01 | -0.05, 0.02 | 0.11 | 0.72 | 0.472 | 0.810 |
| Medial orbitofrontal | -0.01 | -0.04, 0.02 | 0.32 | 0.71 | 0.476 | 0.810 |
| Superior temporal | 0.008 | -0.02, 0.04 | 0.46 | 0.61 | 0.545 | 0.816 |
| Accumbens-area | -0.008 | -0.04, 0.02 | 0.25 | 0.47 | 0.636 | 0.899 |
| Pericalcarine | 0.006 | -0.03, 0.04 | 0.23 | 0.36 | 0.721 | 0.943 |
| Banks of superior temporal sulcus | 0.005 | -0.03, 0.04 | 0.26 | 0.28 | 0.776 | 0.943 |
| Insula | -0.004 | -0.03, 0.02 | 0.49 | 0.28 | 0.782 | 0.943 |
| Putamen | -0.003 | -0.03, 0.03 | 0.36 | 0.23 | 0.818 | 0.957 |
| Rostral anterior cingulate | -0.004 | -0.04, 0.03 | 0.25 | 0.22 | 0.829 | 0.957 |
| Pallidum | -0.003 | -0.03, 0.03 | 0.32 | 0.20 | 0.840 | 0.957 |
| Inferior parietal | 0.002 | -0.03, 0.03 | 0.44 | 0.16 | 0.870 | 0.961 |
| Precuneus | -0.001 | -0.03, 0.02 | 0.51 | 0.10 | 0.917 | 0.961 |
| Inferior temporal | -0.002 | -0.03, 0.03 | 0.43 | 0.10 | 0.917 | 0.961 |
| Frontal pole | 0.001 | -0.03, 0.03 | 0.18 | 0.09 | 0.930 | 0.961 |
| Caudal middle frontal | -0.001 | -0.03, 0.03 | 0.30 | 0.06 | 0.949 | 0.961 |

β, standardized coefficients; CI, confidence interval; FDR, false discovery rate.

## Supplementary Figures


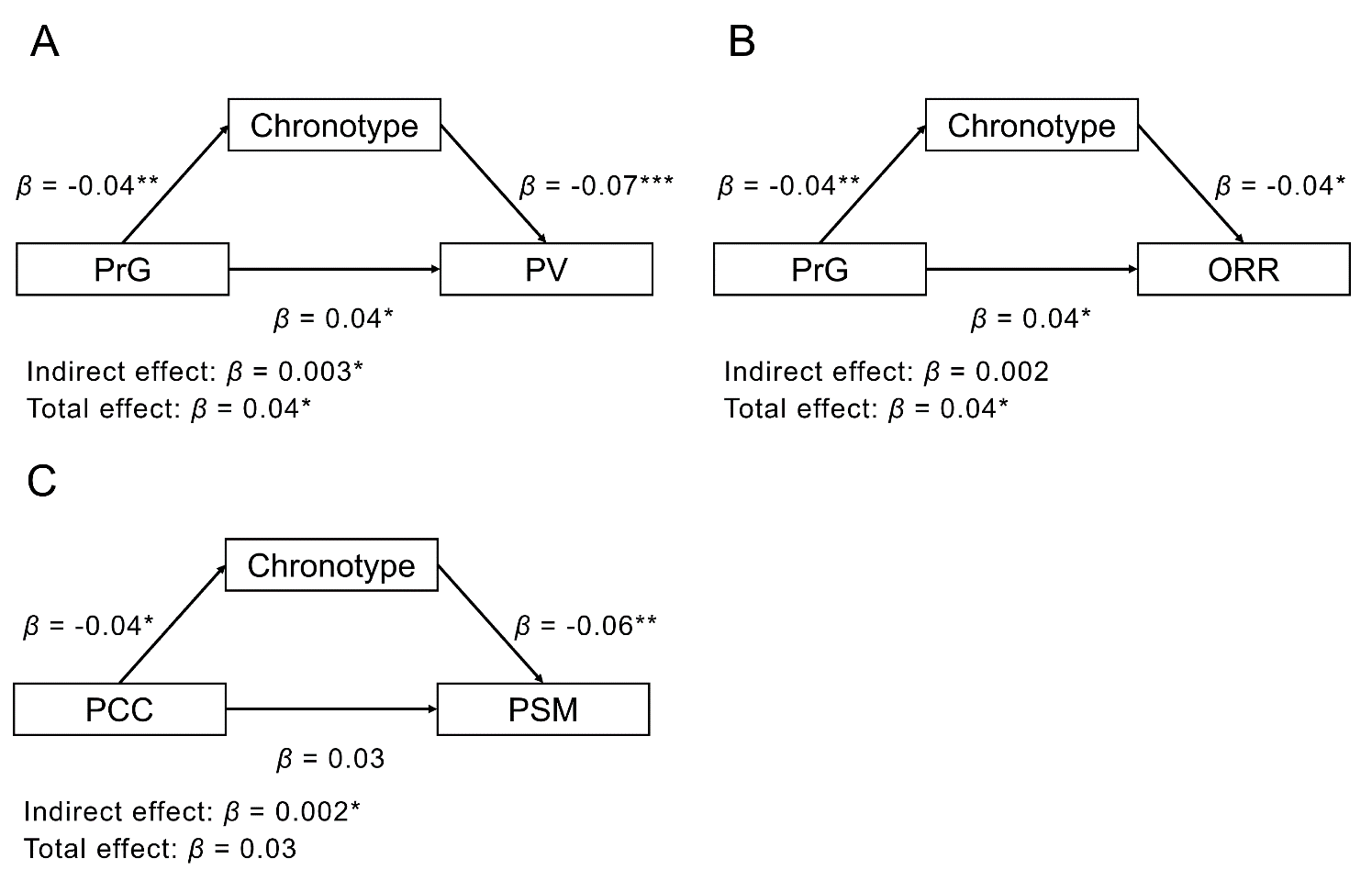


**Supplementary Figure 1.** The mediating effect of chronotype on the association between region-of-interest volumes and language and episodic memory performances. * *P* < 0.05, ** *P* < 0.01, *** *P* < 0.001. PrG, precentral gyrus; PV, picture vocabulary; ORR, oral reading recognition; PCC, posterior cingulate cortex; PSM, picture sequence memory.

# Supplementary References

Bernanke, J., Luna, A., Chang, L., Bruno, E., Dworkin, J., and Posner, J. (2022). Structural brain measures among children with and without ADHD in the Adolescent Brain and Cognitive Development Study cohort: a cross-sectional US population-based study. *Lancet Psychiatry* 9, 222-231. doi: 10.1016/s2215-0366(21)00505-8

Heeringa, S., and Berglund, P. A. (2020). A guide for population-based analysis of the adolescent brain cognitive development (ABCD) study baseline data. *Preprint at https://doi.org/10.1101/2020.02.10.942011.*

Luciana, M., Bjork, J. M., Nagel, B. J., Barch, D. M., Gonzalez, R., Nixon, S. J., et al. (2018). Adolescent neurocognitive development and impacts of substance use: Overview of the adolescent brain cognitive development (ABCD) baseline neurocognition battery. *Dev. Cogn. Neurosci*. 32, 67-79. doi: 10.1016/j.dcn.2018.02.006

Owens, M. M., Allgaier, N., Hahn, S., Yuan, D., Albaugh, M., Adise, S., et al. (2021). Multimethod investigation of the neurobiological basis of ADHD symptomatology in children aged 9-10: baseline data from the ABCD study. *Transl. Psychiatry* 11, 64. doi: 10.1038/s41398-020-01192-8
